# The FlyCatwalk: A High-Throughput Feature-Based Sorting System for Artificial Selection in *Drosophila*

**DOI:** 10.1534/g3.114.013664

**Published:** 2015-01-02

**Authors:** Vasco Medici, Sibylle Chantal Vonesch, Steven N. Fry, Ernst Hafen

**Affiliations:** * Institute of Molecular Systems Biology, ETH Zurich, 8093 Zurich, Switzerland; † SciTrackSs GmbH, Pfaffhausen, Switzerland

**Keywords:** *Drosophila*, systems genetics, population genetics, artificial selection, automated phenotyping

## Abstract

Experimental evolution is a powerful tool for investigating complex traits. Artificial selection can be applied for a specific trait and the resulting phenotypically divergent populations pool-sequenced to identify alleles that occur at substantially different frequencies in the extreme populations. To maximize the proportion of loci that are causal to the phenotype among all enriched loci, population size and number of replicates need to be high. These requirements have, in fact, limited evolution studies in higher organisms, where the time investment required for phenotyping is often prohibitive for large-scale studies. Animal size is a highly multigenic trait that remains poorly understood, and an experimental evolution approach may thus aid in gaining new insights into the genetic basis of this trait. To this end, we developed the FlyCatwalk, a fully automated, high-throughput system to sort live fruit flies (*Drosophila melanogaster*) based on morphometric traits. With the FlyCatwalk, we can detect gender and quantify body and wing morphology parameters at a four-old higher throughput compared with manual processing. The phenotyping results acquired using the FlyCatwalk correlate well with those obtained using the standard manual procedure. We demonstrate that an automated, high-throughput, feature-based sorting system is able to avoid previous limitations in population size and replicate numbers. Our approach can likewise be applied for a variety of traits and experimental settings that require high-throughput phenotyping.

Many traits in natural populations are quantitative; they show approximately Gaussian distributed phenotypic values and are controlled by genetic variation at multiple loci across the genome (Falconer and Mackay 1996; Lynch and Walsh 1998). Our understanding of the causal relationships between genetic variation and quantitative phenotypic variation remains incomplete for most traits, in part due to their highly multigenic nature. The traditional “organism minus one gene” approach has proven to be very powerful for gaining a mechanistic understanding of the functions of individual gene products and their effects on a phenotype. The single gene approach is, however, limited by its ignorance toward environmentally or genetically context-dependent effects of genes and by its bias for large effects; small effects are often overlooked or difficult to reproduce. Given that most quantitative traits are influenced by a large number of often highly context-dependent loci, which by themselves affect the phenotype only a little, a more global approach is evidently needed to obtain a more complete understanding of the genetic architecture of quantitative traits (Falconer and Mackay 1996; Lynch and Walsh 1998; Mackay *et al.* 2009; Huang *et al.* 2012).

Experimental evolution represents an example of such an approach (Zeyl 2006; Bennett and Hughes 2009; Burke and Rose 2009; Kawecki *et al.* 2012). Although laboratory evolution experiments have previously been widely applied to study the mechanisms underlying the evolution of traits and the adaptation of populations to new environments, they can be powerful tools for elucidating the genetic networks underlying quantitative trait variation. Applying artificial selection to laboratory populations generates highly divergent extreme populations for the phenotype of interest. The selected populations can be pool-sequenced to identify alleles that are present at significantly different frequencies in the divergent populations. A difference in the frequency of an allele between the extreme populations hints at a role of the locus in controlling trait variation.

Ideally, the artificial selection process affects only loci that are causally involved in the expression of the trait in question. In reality, however, the majority of enriched variants are false-positives in the sense that neutrally evolving loci can become enriched merely by chance through random processes such as genetic drift. A powerful experimental design should aim at maximizing the proportion of causal loci *vs.* random noise in the selected populations.

An increase in population size and a larger number of replicates correlate positively with a higher proportion of causal (true positive) loci relative to noise (Kofler and Schlötterer 2014). The upper limit on both factors is given by the time and labor available for sampling and phenotyping the population. Consequently, large population sizes and many replicates can only be achieved in evolution studies with simple maintenance and rapid phenotyping. In less complex organisms like bacteria or yeast, propagation is straightforward and selection and phenotyping are often partly automated, enabling high throughput and very large population sizes (Wang *et al.* 2014; Jang *et al.* 2014; Nicoloff *et al.* 2007; Wiser *et al.* 2013). In higher organisms, however, phenotyping is more time-consuming and has so far been the major limiting factor for experimental throughput. Thus, partial or complete automation of the phenotyping step would open up new experimental possibilities for higher organisms, such as *Drosophila melanogaster*, which represents a powerful model organism for experimental evolution experiments (Burke and Rose 2009). *Drosophila* has a short generation time, can be easily maintained in the laboratory, and there are public genomic resources. Additionally, *Drosophila* has certain attractive genomic features for artificial selection, such as a manageable genome size, a rapidly decaying linkage–disequilibrium structure, and high intraspecific genetic variation (Mackay *et al.* 2012).

Artificial selection has been successfully applied to several complex behaviors of *Drosophila* that are amenable to relatively high-throughput selection by partially automating the phenotyping process (Dierick and Greenspan 2006; Edwards *et al.* 2006; Mackay *et al.* 2005). Morozova *et al.* (2007) used an “inebriometer” to quantify alcohol tolerance in a selection experiment for *Drosophila* alcohol sensitivity and effectively identified genes underlying alcohol tolerance [Hirsch developed a maze for easy phenotyping of *Drosophila* phototaxis and gravitaxis, which has been applied in selection experiments for positive and negative geotaxis and phototaxis (Hirsch 1959; Hadler 1964; Hirsch and Erlenmeyer-Kimling 1962)].

In contrast, large-scale studies on size variation in *Drosophila* populations have so far been limited by the prohibitive time investment for phenotyping. There is extensive knowledge about the evolutionarily conserved signaling pathways that control developmental processes from a wealth of single-gene analyses over the past 30 years (Johnston and Gallant 2002; Mirth and Riddiford 2007; Shingleton 2010; Oldham *et al.* 2000). These identified two pathways as the main underlying regulators of size, the Insulin/TOR and the Hippo pathway (Oldham and Hafen 2003; Tumaneng *et al.* 2012; Pan 2007). However, the complete picture is still missing. Because size is clearly a highly multigenic trait (Lango-Allen *et al.* 2010; Gockel *et al.* 2002), evolve and re-sequencing approaches are promising to reveal further insights into the genetic architecture underlying this trait. Unfortunately, manual measurements have so far remained the gold standard for morphometric quantification (Partridge *et al.* 1999; Trotta *et al.* 2007). As a consequence, most published selection studies use population sizes of approximately 10–50 individuals per generation, which is clearly in the lower range and leads to underpowered experimental designs (Kofler and Schlötterer 2014). Houle *et al.* (2003) developed a phenotyping device for *Drosophila* wings that enables live fly wing quantification with significant improvement in speed compared with current techniques but still involves considerable manual labor. Furthermore, it is restricted to wings; other morphometric traits like thorax or head size need to be quantified separately. Another study made use of a sieving apparatus, which enables screening 1800 flies simultaneously for size (Turner *et al.* 2011). Although fast and ingenious, this approach is inaccurate because flies are randomly oriented when passing through the sieve and outstretched legs or wings may hinder an otherwise small fly from passing. Likewise, it does not take into account the size of individual body parts. A fly could end up in the big population due to a big thorax, abdomen, or big wings, which clearly make a difference for subsequent analysis. We posit that an automated phenotyping and selection system for *Drosophila* size traits would be highly beneficial, because it enables a more powerful experimental design of artificial selection studies for morphometric traits by decreasing the phenotyping effort.

An automated morphometric phenotyping system should increase speed while maintaining a level of accuracy comparable with the current gold standard. Applying selection after phenotyping requires single individual quantification and storage, because population statistics are necessary to determine which individuals are selected. We developed a system for the rapid phenotyping of *Drosophila* morphometric traits that meets these requirements: the FlyCatwalk. With a throughput of one fly every ∼40 sec, our system is able to quantify several morphometric features simultaneously. For a set of flies measured both manually and with the FlyCatwalk, we achieve a very good correlation of measurements, thus demonstrating the high accuracy of the automated system. Furthermore, the FlyCatwalk is able to distinguish between males and females and allows storing flies individually until the morphometric analysis is complete. To be able to select specific flies from the measured population, we additionally implemented an automated sorting mechanism.

In summary, we present an automated phenotyping system for *Drosophila* morphometric traits that allows performing extremely time-consuming artificial selection experiments by increasing experimental throughput approximately four-fold while preserving data quality comparable with standard manual measurements.

## Materials and Methods

### Animals

Adult wild-type flies from an outbred population of 176 round-robin mated DGRP lines (Mackay *et al.* 2012) were used for all validation experiments.

### FlyCatwalk workflow

#### Measurement framework:

The FlyCatwalk software consists of a state machine running in Labview 12 (National Instruments Corporation, Austin, TX) that automatically manages the workflow and integrates the different hardware components with the image processing software. We use an Arduino Uno R3 board (Arduino SA, Chiasso, Switzerland), which allows controlling up to six servo motors, four solenoid valves, and six light barriers and communicates with the computer using a USB serial connection. Image processing entails two steps: a fast real-time analysis for image validation followed by a detailed and more time-consuming morphometric analysis (Matlab R2013a; The Mathworks Inc, Natick, MA).

#### Measurement workflow:

**Figure 1:**
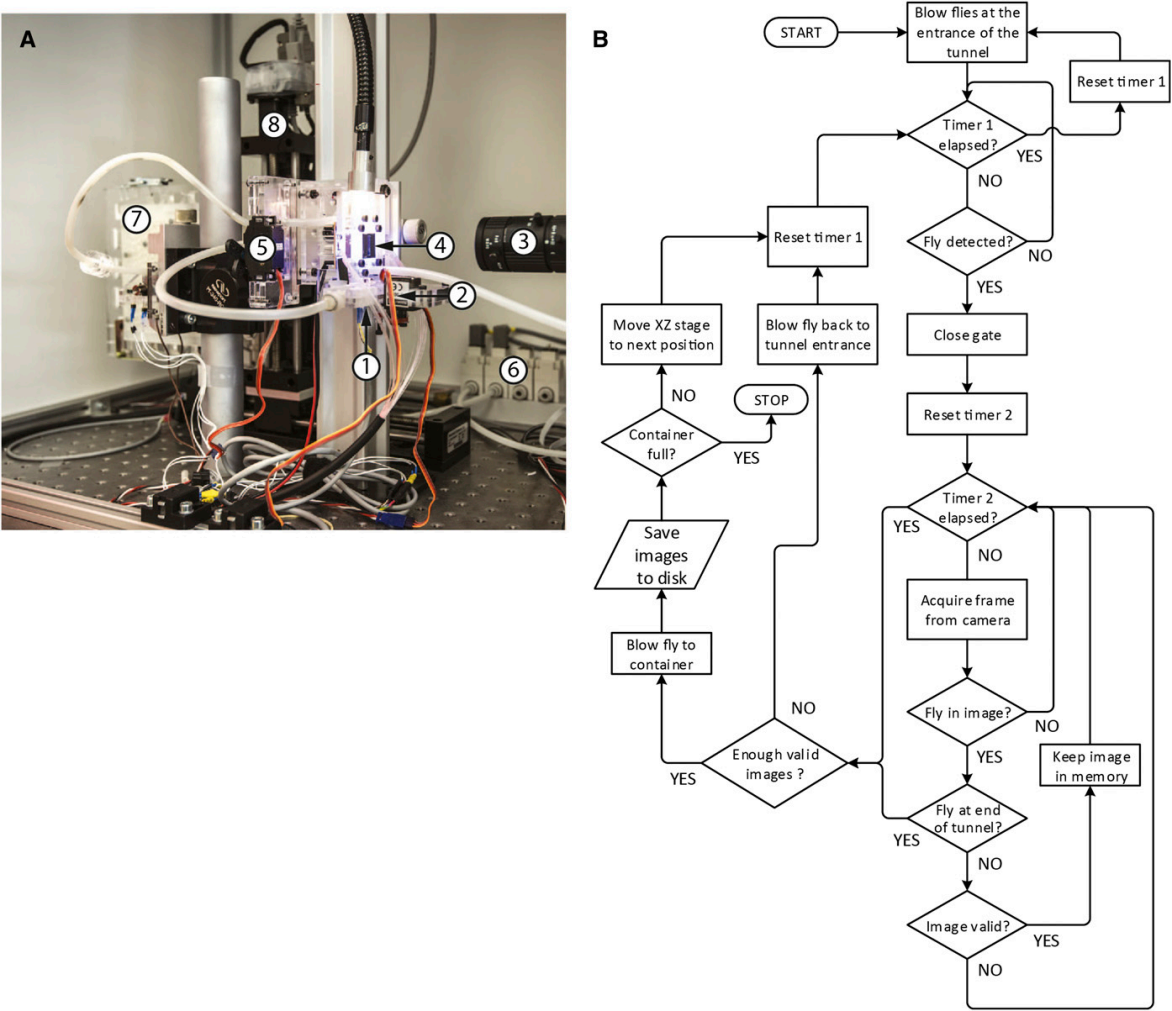
The FlyCatwalk framework and workflow. (A) Setup: 1, tunnel entrance; 2, singling out gate; 3, camera; 4, measurement channel; 5, sorting gate; 6, pneumatic valves; 7, storage device; and 8, XZ robot. (B) Flies in the entrance chamber are regularly activated by air pulses to encourage movement toward the measurement chamber. Once a fly enters the measurement chamber, a gate closes and prevents further flies from entering. While the fly is walking along the tunnel of the chamber, it is imaged with a high-resolution camera and valid images are kept. On reaching the end of the tunnel, the fly is either blown back to the entrance chamber, if too few images are judged valid for analysis, or transferred into a well of the storage device, which then moves one slot further. All images corresponding to stored flies are saved to a disk for in-depth morphometric analysis. There is a time limit for how long a fly may take to cross the channel. If this is exceeded without having acquired sufficient valid images, then the fly is directly blown back to the entrance chamber. The whole process is repeated until all wells of the storage device are filled with flies.

The workflow of the FlyCatwalk is illustrated in Figure 1, A and B. Approximately 60 flies are introduced into the FlyCatwalk entrance chamber. A pneumatic system consisting of a two-port solenoid valve (VQ21M1-5YO-C6-Q; SMC Corporation, Tokyo, Japan) and a system of gates operated with servo motors (Modelcraft WG90MG and Modelcraft MC-965DMG; Conrad Electronic SE, Hirschau, Germany) allows singling out and storage of the measured flies. Flies are activated by air pulses to move toward the measurement channel, a narrow vertical tunnel, that they ascend due to their naturally negative geotaxis (Hirsch 1959; Beckingham *et al.* 2005; Toma *et al.* 2002). Positive phototaxis (Hadler 1964) is also exploited by placing a white light (Ace I; Schott AG, Mainz, Germany) at the far end of the tunnel. At the tunnel entrance, a light barrier detects the presence of a fly and closes a gate behind it. While walking up the tunnel, the singled out fly is imaged by a high-resolution color camera (Basler Pilot piA2400-12gc; Basler AG, Ahrensburg, Germany) at 20 frames per second. The measurement channel is covered by a standard glass coverslip that permits high image quality for filming but can easily be replaced when stained. In the measurement chamber, a two-color illumination strategy is used to image the wings. Blue light provides backlighting where wings do not overlap with the body. A diffusive screen is placed on the tunnel floor to provide homogenous backlighting. The body itself is used as a diffusor to image wing parts that do overlap the body. This is achieved by illuminating the fly from both sides with red light (LXHL-LD3C red high power led, wavelength 627 nm; Quadica Developments Inc, Brantford, Canada) channeled by 22 optical fibers. Using different light channels (blue and red) allows separate analysis of the two channels, for instance, when only the silhouette of the fly is required (as for analyzing body morphology) only the blue channel is used. The absence of direct light from above avoids reflections from the wings. To minimize heat while maximizing light intensity, both light sources are flashed synchronously with the camera exposure with a flash duration of 400 us (0.8% duty cycle) using a strobe controller (Gardasoft PP520F; Gardasoft Vision Ltd, Cambridge, England).

Although the fly is walking up the tunnel, the acquired frames are analyzed in real time to assess image quality and to verify the orientation of the fly. The real-time image processing is performed with a custom-written C++ library, which uses the OpenCV library (Bradski 2000) to perform fast image analysis. For each acquired frame, the following quality checks are performed: image is in focus; body and wings do not touch the image border; no direct reflections from the wings; fly is walking with its wings facing the camera; longitudinal body axis is aligned with the tunnel; and body and wing symmetry match along the body’s longitudinal axis. If all these criteria are met, then the frame is retained and added to a sequence of valid images. When the fly reaches the end of the imaged region, the acquired sequence is analyzed and a decision is made whether to accept or reject the fly based on the number of valid images. If accepted, the fly is blown into a slot of the storage device, where it will be kept until the entire sample population is collected and the in-depth analysis is completed. If the acquired image sequence does not contain enough (≥3) valid frames, the fly is blown back to the entrance chamber to be re-measured.

The storage device consists of a rack of 182 wells 6 mm in diameter and 15 mm deep, each equipped with an independent door mechanism that is automatically opened when a fly is inserted or ejected. The fly container is mounted on a motorized XZ stage (two Newmark ET-150-21 mounted in XZ configuration; Newmark Systems Inc, Rancho Santa Margarita, CA), which is controlled using a two-axes USB controller (Newmark NSC-A2L; Newmark Systems Inc, Rancho Santa Margarita, CA) to allow alignment of the container wells with the measurement channel outlet for single individual storage.

### Data analysis

The data analysis software extracts morphometric measurements for body segments and wings and simultaneously detects the gender. Head, thorax, and abdomen dimensions, interocular distance, and wing morphology are calculated.

### Body segments extraction

The entire image sequence is scanned to first segment the body into head, thorax, and abdomen. The main image processing steps are illustrated in Supporting Information, Figure S1. The blue color channel is used to extract the silhouette of the fly for body segmentation. Each frame is subtracted from the background, which results in a bright fly against a dark background. This image is inverted to generate the complement image, a dark fly on a bright background. Only the frames that were considered valid during the acquisition are used. Using the central image moments, the centroid and main axis are extracted from the complement images and used to align the single frames in a stack (Figure S1A). A 95^th^ percentile image is then calculated from the stack to delete the legs, which are constantly moving while the fly is walking and therefore exposes the background underneath (Figure S1B). The 95^th^ percentile image is thresholded and an image closing morphological operation (Serra 1983) is applied to remove thin structures, such as wing veins and bristles. The three body segments are extracted from the obtained binary image using the watershed segmentation algorithm (Roerdink and Meijster 2000) (Figure S1C). The dimensions of each segment are subsequently evaluated by fitting templates to their contours.

### Sex discrimination

**Figure 2:**
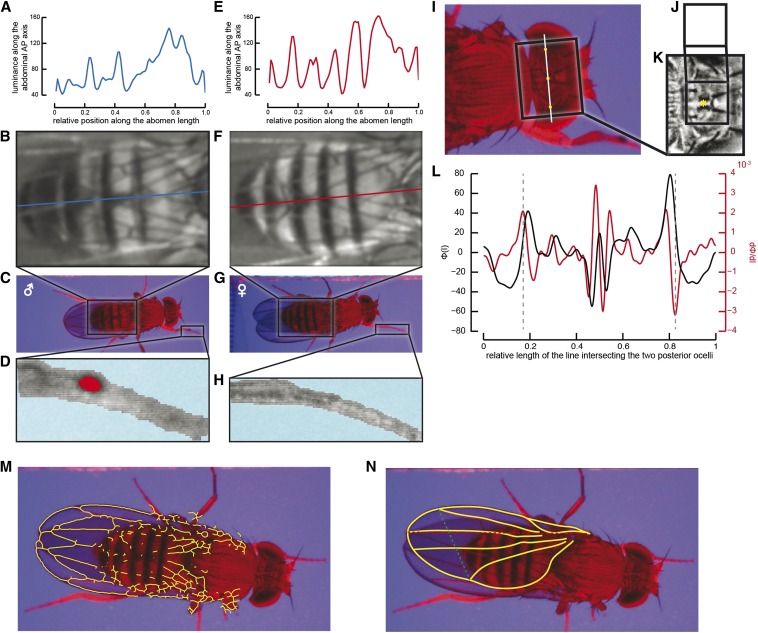
Morphometric analysis. (A–E) sex detection. Sex is determined using two methods. First, the luminance (A, E) is evaluated along the abdomens’ longitudinal axis (B, F) and compared with templates of male and female abdominal luminance to determine which of the two templates gives the better correlation. Second, from the entire video sequence, the most anterior leg pair (C, G) is identified and scanned for the existence of sex combs (D, H), which are only present in males. (I–L) Detection and quantification of interocular distance. The position of the head is determined from the body segmentation (Figure S1). The position of the ocelli is extracted using a template-matching algorithm (J, template; K, result of the template matching algorithm). The luminance (black line in L) is evaluated along the line intersecting the two posterior ocelli (white line in I) and its derivative (red line in L) is computed to identify regions of high contrast (dashed lines in L) corresponding to the border of the compound eyes. (M, N) Wing fitting and quantification. The raw image is filtered and binarized, and subsequently the skeleton extraction algorithm is applied to it to detect vein structures (M). A B-spline is fit to the wing to determine outline and veins L2-L5 (N). Yellow solid lines: B-spline fit of the wing outline and veins L2-L5. Red dashed line: segment used to define wing length. Green dashed line: segment used to define wing width.

Two methods are combined for increased robustness. The luminance in the red channel along the abdomen is first normalized using mean luminance and abdomen size that were extracted from a set of manually sorted measurements and subsequently cross-correlated with average male and female luminance curves (Figure 2, A, B, E, F). The sex of the individual is determined by the curve yielding the higher correlation coefficient. Because luminance patterns may vary between flies depending on their genotypic background or their hydration state, this method is not sufficient for sex discrimination. We therefore implemented a complementary detection method consisting of scanning the single frames for the existence of sex combs. The images are scanned for structures extending anterior to the head along the body longitudinal axis (Figure 2, C and G). The sex combs are then identified by applying a threshold to the leg image in the red channel and scanning for dark spots (Figure 2, D and H). The eccentricity of the detected regions is calculated and objects yielding high eccentricity values are rejected to avoid false-positives when the leg is crossing the antennae. The confidence level of both methods is used to determine sex. For the abdominal luminance method, confidence is estimated based on how different the two correlation coefficients are. For the sex comb method, the confidence level is determined based on an average of the area of the sex combs on the images they were detected on and are based on the proportion of images they were detected on. When the combined results do not yield a clear decision for the sex, the sex is marked as unknown and may be determined by the user in the verification graphical user interface (GUI).

### Interocular distance extraction

The head position is detected on the brightest frame in the red channel by binarizing the background-subtracted image in the blue channel and aligning it with the previously extracted body model (Figure 2I). The ocelli are detected using a template matching method (Figure 2, J and K) and the light intensity along the line intersecting the two posterior ocelli is evaluated (Figure 2L, black line). The eye edges are identified by scanning the derivative of the luminance (Figure 2L, red line) for sharp contrast edges (Figure 2L, dashed lines). Interocular distance is quantified as the length of the line segment crossing the center of the posterior ocelli, from eye edge to eye edge.

### Wing extraction

Wings are detected using a template consisting of the outline and wing veins L2 to L5. Wing length is defined as the length of the segment between the wing hinge and the intersection of the wing outline and vein L3 (Figure 2N, red dashed line). Wing width is measured as the length of the line connecting the intersection of the outline with vein L2 to the intersection of the outline with vein L5 (Figure 2N, green dashed line). The wing-fitting algorithm is implemented in Matlab and fits B-splines to the topological skeleton of the wing outline and veins extracted from the acquired video frames. The brightest frame in the red channel is selected for the procedure to ensure maximum brightness of the abdomen, which allows a sharper contrast between wing and body and facilitates detection of the wings and wing veins in regions where body and wings overlap. The algorithms used to extract the topological skeleton of the wing veins and outline are slightly different between the overlapping and the nonoverlapping regions, but the principles are the same. The blue channel is used for the nonoverlapping regions because the backlighting allows one to clearly see through the wings, whereas for the overlapping regions the red channel is used. Contrast-limited adaptive histogram equalization (Zuiderveld 1994) is applied to the background subtracted image, which allows enhancing the local contrast of the image while minimizing luminance gradients. The image is thresholded and cleaned using a series of morphological operations (Supporting Information, File S1) and the resulting black and white image is skeletonized using the anaskel.m function^4^ (Figure 2, M and N).

### User verification of the analysis outcome

A GUI for visual verification and manual adjustment of the outcome of the automatic analysis was implemented in Matlab. The following parameters can be verified and changed: sex; interocular distance (IOD); wing length (WL); wing width (WW); and wing area (WA). At present, the fit of body segments and the complete wing vein morphology can be visually checked but not modified^5^. Additionally, wells can be marked for exclusion, for example, if the corresponding fly’s wings are damaged or in the rare cases when two flies ended up in one well.

### Sorting

Using the pneumatic system, flies are blown out of the storage wells into a collection container. Currently, sorting is implemented to select for relative wing size (wing size normalized to body size), but other measured phenotypes can be implemented as selected traits. The selection allows sorting the top and bottom numbers of individuals for each sex in terms of relative wing size, whereby the exact number of individuals can be modified by the user in the analysis GUI.

### Control measurements

A total of 147 flies (77 males and 70 females) were measured in the FlyCatwalk and then collected individually and frozen at −20° for manual phenotyping.

### Body

Flies were positioned on a black apple agar plate and photographed with a VHX-1000 digital light microscope (KEYENCE, Itasca, IL). Morphometric body traits were measured manually using the VHX-1000 built-in measurement software. Interocular distance was measured from eye edge to eye edge along the center of the posterior ocelli and parallel to the base of the head. Shoulder width was measured as the distance between the left and right humeral bristles.

### Wings

The intact left or otherwise right wing was dissected from the fly and mounted in water on a glass slide for wing image acquisition. Morphometric measurements were extracted from the wing images using the WINGMACHINE software (Houle *et al.* 2003) and Matlab.

## Results

### The FlyCatwalk: high-throughput automated phenotyping of morphometric traits

We developed the FlyCatwalk (Figure 1, Supporting Information, File S2), a system that enables increased phenotyping throughput while maintaining a measurement accuracy comparable with the current gold standard and has a sorting function for selection experiments. In the FlyCatwalk, flies are singled out, imaged while walking through a measurement chamber, and subsequently collected individually in different wells of a storage device. With the FlyCatwalk, we can measure multiple morphometric traits simultaneously: wing area, wing length, and wing width; the dimensions of the thorax, abdomen, and head; and two measures that we chose as proxies for body size, interocular distance and shoulder width. Other measures can easily be implemented based on the body segment dimensions.

The FlyCatwalk consists of three modules, the entrance chamber, the measurement tunnel, and the storage device, which are interconnected with plastic tubes for transferring flies between the modules using a pneumatic system (Figure 1, A and B). Cold-anesthetized flies need to be loaded approximately 60 at a time into the entrance chamber, where timed air pulses animate them to enter the measurement tunnel. A gating mechanism ensures that flies are singled out at the entrance. Once in the measurement chamber, each fly is imaged by a high-resolution camera at 20 frames per second while walking vertically through the tunnel. We apply some quality filtering criteria to evaluate whether an image is valid, the most important of which requires that the fly be oriented with its wings facing the camera. A fly is kept and transferred to the storage device when at least three images are valid, the minimum required for subsequent in-depth morphometric analysis. Otherwise the fly is blown back to the entrance chamber. This cycle is repeated until all storage wells are full. The user occasionally needs to reload flies but otherwise is free to leave, as the FlyCatwalk is fully self-operating. Flies do not need to be sorted by sex prior to phenotyping because the analysis software is able to distinguish between males and females.

We store the flies individually until the in-depth morphometric analysis is complete. Individual storage has the advantage that specific flies from the population can be identified and further used for experiments or breeding. For a selection experiment, it is, for instance, necessary to know the phenotypes of all individuals in a population before the largest 20% can be determined. Using the pneumatic system of the FlyCatwalk, these can be specifically sorted from the storage device to form the parents of the next generation. In summary, the FlyCatwalk enables live phenotyping of multiple traits simultaneously with minimal user intervention and offers additional functionalities that may be useful for selection studies.

### Experimental throughput

To determine the realized increase in phenotyping throughput of the automated system, we compared the per-day phenotyping performance of our system with that of a standard method, *i.e.*, manual quantification. With the FlyCatwalk, we currently achieve a throughput of approximately one fly every 40 sec, which yields a total throughput of 720 flies per 8-hr day. Based on experience, this number is variable depending on the sex of the flies and the time of the day. Throughput is higher for males, which could be a consequence of higher intrasex aggression behavior, and in the morning and evening compared with throughout the day, which reflects the natural activity pattern of *Drosophila* (Allada and Chung 2010; Klarsfeld *et al.* 2003). Nevertheless, we routinely achieve a throughput of more than 500 mixed-sex flies per day. Using our optimized manual phenotyping procedure, which involves one person positioning and a second person photographing the flies, we can currently process 250 flies per day, but we need an additional day for acquiring all morphometric measurements. The FlyCatwalk therefore offers an approximately four-fold increase in phenotyping throughput compared with manual processing while reducing the labor required to one person. Beyond the phenotyping functionality, the FlyCatwalk is able to sort flies from the measured population based on user-defined selection criteria, a process that is very cumbersome to do manually, and thus additionally reduces the time and labor investment for selection experiments.

### Morphometric analysis

We describe the morphometric analysis in detail in the *Materials and Methods* and in Figure 2 and Figure S1. Briefly, the data analysis software extracts morphometric measurements for body segments and wings and simultaneously detects the gender. The body is first segmented into head, thorax, and abdomen, and the dimensions of the individual segments are determined (Figure S1). The sex is detected by combining two methods: quantification of the luminance along the abdominal anterior–posterior axis (Figure 2, A, B, E, F) and detection of sex combs (Figure 2, C, D, G, H). The software calculates the interocular distance based on the head model, the ocelli, and the eye edges (Figure 2, I–L), thus using the same landmarks as in manual quantification. Shoulder width, in contrast, is extracted indirectly from the scaling parameter applied to the thorax template by the template-matching algorithm and not by detecting the humeral bristles, which are the landmarks used for manual phenotyping. A template consisting of the wing outline and veins L2 to L5 is fitted to the wings, and then wing length, wing width, and wing area are calculated (Figure 2, M and N). Even if in principle the system does not require it, a visual verification of the data is always desirable. It not only gives the user control over the accuracy of the fitting but also allows discarding flies with damaged wings. For this reason we provide a Matlab GUI that allows visual verification and manual adjustment of the analysis.

### Method validation

**Figure 3:**
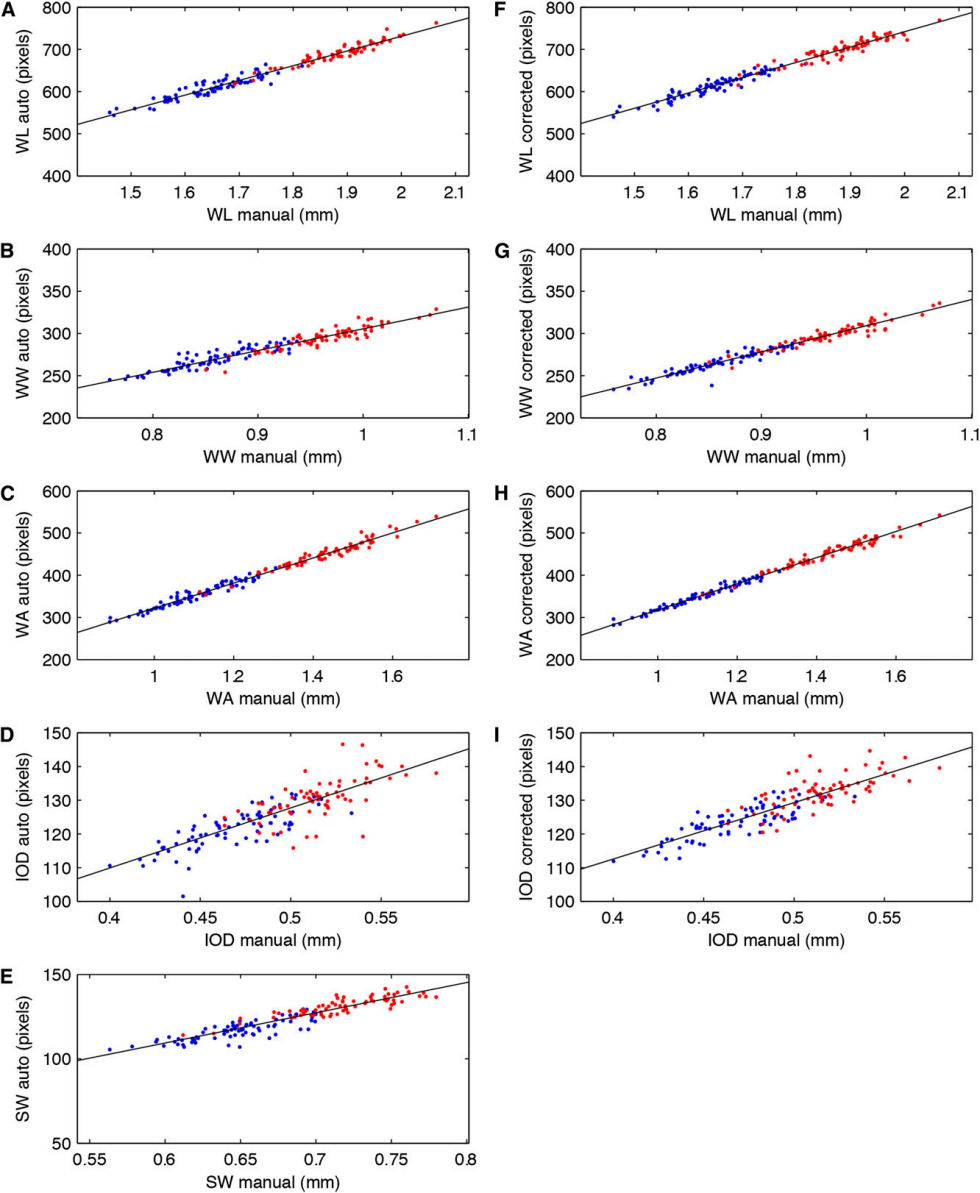
Automated and manually acquired measurements are highly similar for wing traits and show good correlation for body traits. Correlations between measurements acquired with the manual (x-axis) and the automated (y-axis) methods are shown (A–E). The correlation is very high for all wing traits, with wing area being most similar between the two methods, showing the high accuracy of the automated wing phenotyping (A–C). Correlation is somewhat lower for the body traits (D, E), reflecting the difficulty of accurately quantifying 3D objects in a 2D plane. Blue dots, males; red dots, females; black lines, result of robust bi-square linear regression fit through the data; N = 147. (F–I) Same manual data plotted against user-adjusted automated data demonstrate no noticeable improvement in the correlation between methods.

**Figure 4:**
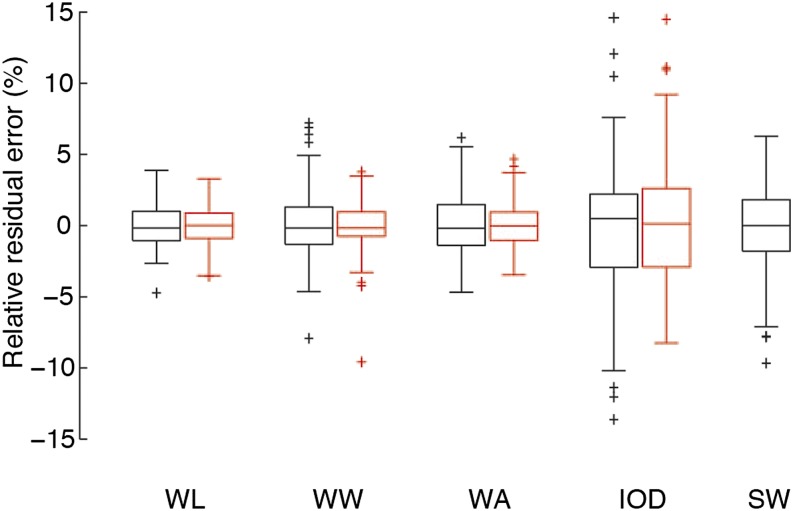
User adjustment does not significantly improve the relative accuracy of the automated method. Residual errors of the robust linear regression are plotted. The error distribution is tight (WL, 0.06 ± 1.56%; WW, 0.09 ± 2.39%; WA, 0.06 ± 2.05%) for all wing traits with the bulk of the data within the 5% deviation range, whereas IOD and SW error distributions are broader (IOD, −0.35 ± 4.83%; SW, −0.15 ± 2.74%) and contain more outliers (black). User adjustment of the automated fitting does not notably reduce the relative error (WL, −0.04 ± 1.46%; WW, −0.09 ± 1.61%; WA, 0.02 ± 1.53%; IOD, 0.16 ± 4.04%), indicating that discrepancies do not arise due to inaccuracies of the automated phenotyping algorithms. There is a slightly beneficial effect of adjustment visible in the shifts of the median WL, WA, and IOD errors toward 0 (red). Parameters of the distributions are given as mean ± SD in % of the manually measured value. On each box, the central line marks the median, the edges of the box mark the 25^th^ and 75^th^ percentiles, and the whiskers mark the most extreme data points not considered outliers (median [first and third quartiles] before correction: WL, −0.17 [−1.07, 1.00]; WW, −0.17 [−1.32, 1.30]; WA, −0.19 [−1.41, 1.48]; IOD, 0.49 [−2.94, 2.20]; SW, −0.00 [−1.81, 1.82]; and after correction: WL, 0.02 [−0.90, 0.87]; WW, −0.18 [−0.76, 0.96]; WA, −0.02 [−1.04, 0.94]; IOD, 0.12 [−2.91, 2.61]). Outliers are defined as being more than 1.5 box-widths smaller or larger than the 25^th^ or the 75^th^ percentiles, respectively. Black boxplots, automatic quantification data; red boxplots, manually adjusted automatic quantification data; N = 147.

Increasing throughput through automation often entails a decrease in measurement accuracy. To this end, we evaluated the accuracy of the automatic image processing compared with manual handling, which represents the current gold standard for morphometric phenotyping. We chose five test phenotypes that may be of interest for morphometric studies and that derive from different body parts. The WL, WW, and WA represented the wing phenotypes, whereas for the head we selected IOD and for the thorax we selected shoulder width (SW). We observed high correlation between manual and automated measurements for all wing traits (R_WL_ = 0.98, R_WW_ = 0.95, R_WA_ = 0.99) and slightly lower correlation for the two body traits (R_IOD_ = 0.83, R_SW_ = 0.93) (Figure 3, A–E), which may reflect the difficulty of quantifying 3D objects accurately from 2D projections. To estimate the relative error of the automated method, expressed in percent of the manually measured data, we applied bi-square robust linear regression and analyzed the residuals (Figure 4, black boxes). This clearly shows that while the errors for all wing traits, with the exception of a few outliers, do not exceed 5%, the error distributions are wider for the body traits (WL, 0.06 ± 1.56%; WW, 0.09 ± 2.39%; WA, 0.06 ± 2.05%; IOD, −0.35 ± 4.83%; SW, −0.15 ± 2.74%). To determine whether these errors arose due to inaccuracies of the automatic processing, we ran the same analysis after the results of the automated fitting had been user-corrected with the verification GUI. Adjustment resulted in a moderate improvement of the correlation coefficients for WW and shoulder width (R_WL_ = 0.98, R_WW_ = 0.98, R_WA_ = 0.99, R_IOD_ = 0.87) (Figure 3, F–I), and for the wing traits in slightly tighter error distributions (WL, −0.04 ± 1.46%; WW, −0.09 ± 1.61%; WA, 0.02 ± 1.53%) (Figure 4, red boxes). Although the error distribution of IOD did not change considerably in broadness (IOD, 0.16 ± 4.04%), we observed a shift of the median toward 0, indicating a moderately beneficial effect of adjustment. Because the current version of the verification software does not include SW correction, we did not correct this trait manually. In general, user correction of the fitting on the acquired images did not improve the quality of the correlation coefficients in a substantial manner, suggesting that the inconsistencies between the manual and automatic measurements do not arise from the image processing and template fitting algorithms, but instead could be a consequence of differences in flies’ body and wing posture between the two methods.

## Discussion

We present an automated system for the rapid phenotyping of *Drosophila* morphometric traits that allows performing artificial selection experiments with a substantially increased throughput than was previously possible while preserving data quality comparable with standard manual methods.

### Novelty and relevance

A key factor for the design of powerful selection experiments are large population sizes, which have so far been limited to only tens of individuals in *Drosophila* morphometrics studies due to the highly time-demanding phenotyping process. By automating the phenotyping using the FlyCatwalk, we can greatly decrease the per-fly time investment and thus achieve population sizes that far exceed the past standard. The application of the FlyCatwalk is not restricted to selection experiments; instead, we consider it a valuable tool in all studies requiring intermediate to high-throughput phenotyping or sorting of *Drosophila melanogaster* and other species based on morphometric features.

To our knowledge, this is the first fully automated large-scale method for the analysis of size traits in *Drosophila*. The FlyCatwalk is able to quantify several aspects of *Drosophila* adult morphology simultaneously with high accuracy, and the spectrum of phenotypes can even be extended. With the FlyCatwalk, we routinely phenotype 500 flies per day, although the maximum throughput could be as high as ∼700 flies/d. We thus estimate that our system provides at least a four-fold increase in phenotyping throughput compared with manual processing while reducing the labor required of one person.

A further major advantage of the FlyCatwalk is the possibility of measuring flies alive. Live-fly phenotyping greatly simplifies selection protocols by enabling phenotypic analysis before breeding. In selection experiments with manual phenotyping, which is simpler on dead flies, many pairs of males and females have to be mated prior to phenotypic analysis, but only the progeny of the phenotypically most extreme pairs will be kept to form the next generation. This strategy requires more time, resources, and space because many crossing vials need to be prepared and stored, it may additionally limit the number of flies that can be analyzed for each generation and it effectively limits the population size. There are examples of manual live-fly morphometric phenotyping, but this is often either inaccurate or cumbersome (Houle *et al.* 2003; Turner *et al.* 2011). Automated live-fly phenotyping may also be desirable in other experiments, such as in studies evaluating the influence of fly size on behavioral traits like mating or flying ability.

In summary, we believe that our system represents a valuable tool for various studies addressing growth-related questions in *Drosophila melanogaster*. Especially for experimental evolution of size traits, the FlyCatwalk opens new possibilities by enabling more powerful experimental designs and thus has the potential to contribute to a more complete understanding of animal growth.

### Are the requirements of an automated measurement system fulfilled?

#### Speed:

Phenotyping with the FlyCatwalk is on average at least four-times faster than manual processing. The maximum throughput depends largely on the walking behavior of the flies, which may vary between individuals, sexes, time of day, days, and seasons. Generally, a throughput of between 500 and 700 flies per 8 hr is feasible, with an average of 90 flies processed per hour. A factor that could help keep the throughput higher is a constant temperature of approximately 20° because walking activity markedly decreases if the temperature increases toward 25°. Apart from the improvement in the phenotyping throughput, the FlyCatwalk also reduces the necessary labor to one person, because it is fully self-operating after the flies have been loaded.

#### Measurement accuracy:

The FlyCatwalk enables very precise quantification of wing traits, as is evident from the high correlation between manually obtained and automated measurements. The correlation is lower for the two body morphometric traits, IOD and shoulder width (SW), which is likely a consequence of trying to quantify parts of a 3D structure from a projection onto a 2D image. In contrast to the flat structure of the wing that, due to its venation pattern, naturally offers very defined landmarks as quantification reference points, IOD and SW need to be defined from less distinct landmarks. The distance between the landmarks on the planar projection may vary depending on the flies’ head or thorax posture on the image. As we control posture only in the manual procedure, this may account for the discrepancies between the two methods. The different definitions of SW between the automated and manual method is a further plausible reason for the low observed correlation: the automated SW measure is calculated from the scaling parameter applied to the thorax template by the template-matching algorithm, whereas the manual procedure uses the humeral bristles as reference points. Because both IOD and SW are measures that we routinely use to determine head size and body size manually, we used these traits in the FlyCatwalk to be able to make quantitative comparisons between the manual and automated method. As a future perspective, choosing more robust measures for head or thorax size will result in more accurate quantification. A defined partial area is more suited to machine vision and should be easy to implement because we already detected the full head, thorax, and abdomen shape in the current analysis.

#### Single fly measurement and storage:

Despite the gating system, two flies may occasionally be measured and stored together, but these can be discarded during manual checking by marking the corresponding well for exclusion in the analysis GUI. We match flies to their corresponding phenotypes by coding the images and measurements according to the fly’s location in the storage device. The high percentage of successfully singled out flies and the labeling system thus ensure that the FlyCatwalk meets this requirement.

### Limitations of the FlyCatwalk

The FlyCatwalk is based on voluntary walking behavior, which introduces a bias for measuring the most active flies in the population. We designed the system exploiting the fact that average flies show negative gravitaxis and positive phototaxis. Because there is variability for both these behaviors on the population level, and because they are heritable (Hirsch 1959; Toma *et al.* 2006; Hadler 1964), we preferentially phenotype flies with strong negative gravitaxis and positive phototaxis. Flies that have sustained mutations or injuries that prevent them from walking vertically, in contrast, cannot be measured. We noticed that voluntary walking behavior is strongly dependent on the sex and the time of day. An all-female population takes longer to measure than an all-male one, which could be a consequence of their lower intrasex aggression levels, making them feel more comfortable in the crowded and confined space of the entrance chamber than males. Phenotyping throughput also shows characteristic morning and evening “spikes,” being high in the morning and then markedly decreases throughout the day and increases again in the evening. This reflects the natural activity curve of *Drosophila melanogaster* (Allada and Chung 2010; Klarsfeld *et al.* 2003). On the analysis side, computational time is, at the moment, a major limiting factor for throughput, and code optimization should be performed to increase analysis speed.

In its current state, the system is still in its prototypical version; therefore, it is not prone to be easily replicated. We are currently working on the next version of the FlyCatwalk, which will be well-documented and easier to replicate, and we are committed to release it open source. In the meantime, the drawings, electrical schematics, and code of the current version of the FlyCatwalk are available on github (https://github.com/IMSB/FlyCatwalk/). However, concerning this first version of the code, because it has never been tested on a different platform than the one on which we ran the experiments, we have no experience regarding how difficult it would be to port it to a different hardware or software.

### Applications of the FlyCatwalk beyond selection experiments of *Drosophila melanogaster*

Beyond the currently implemented morphometric traits (IOD, SW, WA, WL, WW), other traits that are based on head, thorax, abdomen, or wing size and shape can easily be added. Furthermore, our setup can be modified and extended to include quantification of other parts of the body, such as the eyes. Many studies of *Drosophila* use the eye disc as a model organ (Leevers *et al.* 1996; Ling *et al.* 2010; Koontz *et al.* 2013; Nowak *et al.* 2013) and require quantification of adult eye size, the usual readout for the amount of growth, and proliferation in the eye disc. All features on the body that are amenable to detection and quantification by machine vision, such as bristles and bristle number, could also be implemented easily. High-throughput quantification of these traits reduces the timespan required for experiments and is especially beneficial for large-scale projects such as forward and reverse genetic screens and genome-wide association studies.

A valuable application of our system beyond studies of morphometric traits could be in the production of transgenic flies, where successful transformants with red eyes have to be sorted from among large numbers of white-eyed flies. Because this is a binary decision based on a single feature, color, this task is ideally suited to machine vision and automation. Furthermore, the FlyCatwalk could be adapted to quantify the response to various stimuli, such as different odors, light, or gravitaxis, and perform selection on this trait.

The present setup is not restricted to *Drosophila melanogaster*; quantification of other species that approximately match its size and weight is possible after minor adjustments in the morphometric fitting, primarily the template. For larger and heavier specimens, adjustments on the modular parts, such as the diameter of the entrance, measurement, and storage compartments, as well as the air pressure, are necessary. As the logic of the setup is the same, this should be straightforward based on the existing design templates. We estimate the current size limit somewhere below the size of *Sepsis thoracica* (4.51 mm length, 1.06 mm width, 1.27 mm thorax height).

These examples show that the Fly Catwalk, in addition to its application in selection studies, represents a valuable tool in a number of experiments, from large-scale morphometry screens and genome-wide association studies in *Drosophila melanogaster* and other species to transgenic fly production or, more generally, in all types of experiments that require high-throughput phenotyping or sorting based on morphometric features.

## Supplementary Material

Supporting Information

## Footnotes

4 Written by Nicholas R. Howe, available at http://www.mathworks.com/matlabcentral/fileexchange/11123-better-skeletonization.

5 The possibility of modifying the wing vein morphology has not been implemented yet, because it was not required for the present study.
