# Supplementary material for: The FlyCatwalk: A High-Throughput Feature-Based Sorting System for Artificial Selection in Drosophila
Source: G3 (Bethesda). 2015 Jan 2;5(3):317–27. doi: 10.1534/g3.114.013664 (PMC4349086; doi:10.1534/g3.114.013664)
Supplement: Supporting Information [file supp_5_3_317__index.html]

The FlyCatwalk: A High-Throughput Feature-Based Sorting System for Artificial Selection in Drosophila — Supporting Information 

# The FlyCatwalk: A High-Throughput Feature-Based Sorting System for Artificial Selection in *Drosophila*

## Supporting Information for Medici *et al.*, 2015

**Files in this Data Supplement:**

- Supporting Information - Figure S1 and Files S1-S2 (PDF, 1 MB)
- Figure S1 - Body segmentation. (PDF, 1 MB)
- File S1 - Supporting Methods (PDF, 134 KB)
- File S2 - Supporting video - The FlyCatwalk setup and workflow. Individual steps of the automated phenotyping and detailed parts of the setup are shown. (.avi, 9 MB)
