# Supplementary material for: The FlyCatwalk: A High-Throughput Feature-Based Sorting System for Artificial Selection in Drosophila
Source: G3 (Bethesda). 2015 Jan 2;5(3):317–27. doi: 10.1534/g3.114.013664 (PMC4349086; doi:10.1534/g3.114.013664)
Supplement: Supporting Information [file supp_g3.114.013664_FigureS1.pdf]

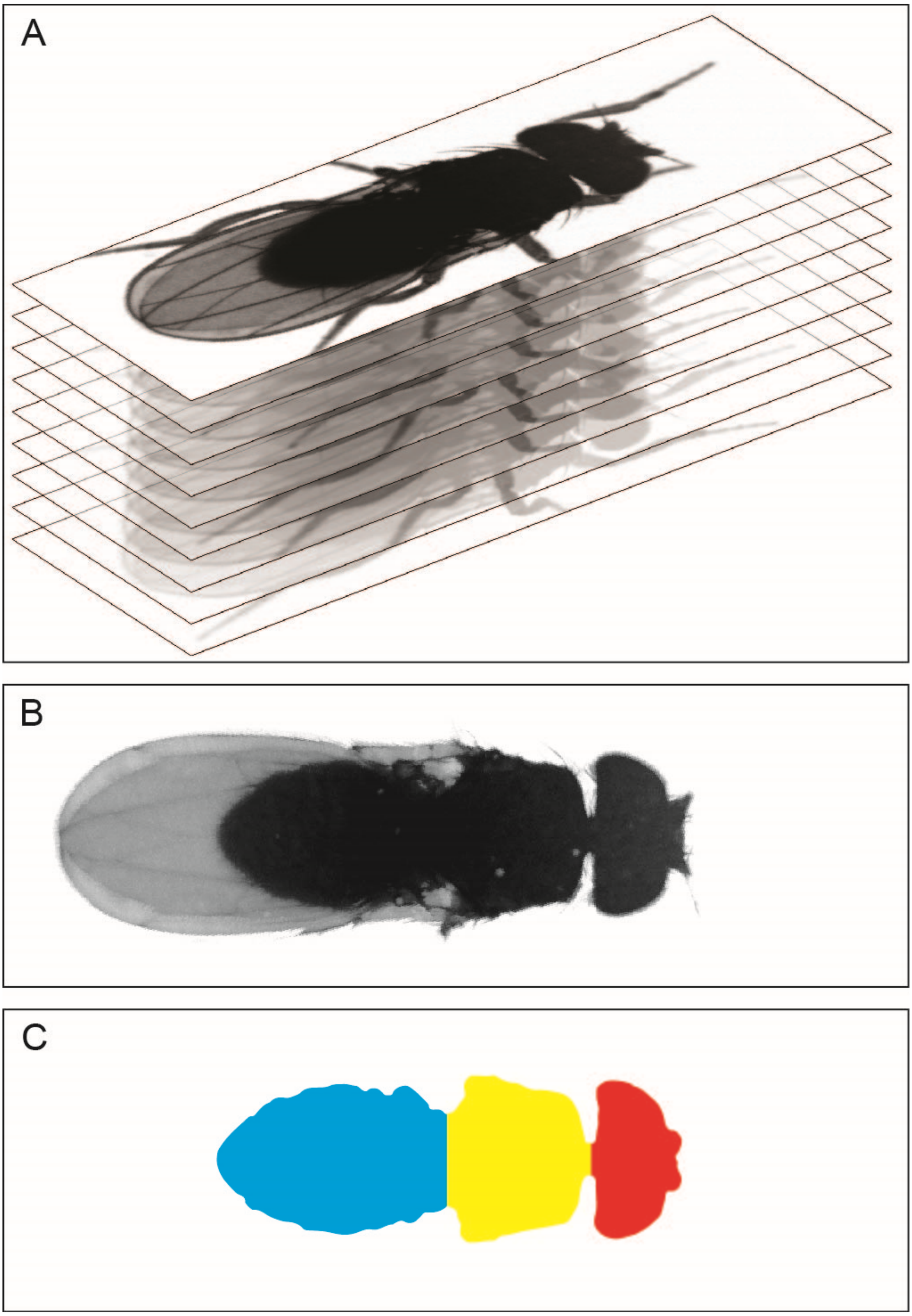

Figure S1 Body segmentation. A: Single complement image frames of the blue channel luminance after background subtraction. Background subtraction results in an image of a bright fly against a dark background. This image is inverted to gain the complement image, a black fly on white background. B: Image after leg deletion. The valid complement frames in the sequence are rotated until they are all aligned and the 95th percentile image is computed to remove the legs, which is possible because their position varies between frames. C: Body segmentation performed using the watershed algorithm (Red= head, yellow = thorax, blue = abdomen).
