# Supplementary material for: The FlyCatwalk: A High-Throughput Feature-Based Sorting System for Artificial Selection in Drosophila
Source: G3 (Bethesda). 2015 Jan 2;5(3):317–27. doi: 10.1534/g3.114.013664 (PMC4349086; doi:10.1534/g3.114.013664)
Supplement: Supporting Information [file supp_g3.114.013664_FileS1.pdf]

File S1

Supporting Methods

Wing extraction

Different approaches are applied for wing regions depending on whether they overlap with the body or not. For the regions without overlap mostly the blue channel is used (names of matlab functions in italics):

1. Image adjustment: image adjustment (*imadjust*) and 2D adaptive noise removal filtering (*wiener2*), 2. Body detection: thresholding followed by image filling and closing to delete holes in the body mask (*imfill and imclose*), 3. Wing detection: High pass filtering of the image to cancel out luminance gradients followed by detection of the wing mask (i.e. selecting a region of interest (mask) in which to apply the image processing). Mask definition is performed using adaptive thresholding techniques and the body masked computed in the preceding step and if present, holes are filled (*imfill*). Legs are detected in the wing mask by additionally including the red channel, in which the legs appear bright, and these regions are removed. This is necessary because legs might get mixed up with veins. 4. Vein detection: A combination of thresholding and edge detection (*edge*, method option 'canny') is used to binarize the image and small objects are deleted (*bwareaopen*). A skeletonization algorithm is applied to extract the structure of the veins (*anaskel*)

For regions with overlap the red channel is used:

1. Selection of the region of interest from the body mask, 2. Image adjustment: Detection of high spatial frequency content using top-hat filtering (*imtophat*) followed by contrast enhancement using contrast-limited adaptive histogram equalization (CLAHE, *adapthisteq*), 3. Binarization and cleaning: Thresholding, image closing to join adjacent blobs and deletion of small objects (*bwareaopen*). Objects with high eccentricity (i. e. they are line-like) are selected. 4. Vein detection: A skeletonization algorithm is applied to extract the structure of the veins (*anaskel*).

File S2

Supporting Video

The FlyCatwalk setup and workflow. Individual steps of the automated phenotyping and detailed parts of the setup are shown.

File S2 is available for download as an AVI file at http://www.g3journal.org/lookup/suppl/doi:10.1534/g3.114.013664/-/DC1
